# Microscale Insight into Microbial Seed Banks

**DOI:** 10.3389/fmicb.2016.02040

**Published:** 2017-01-09

**Authors:** Kenneth J. Locey, Melany C. Fisk, J. T. Lennon

**Affiliations:** ^1^Department of Biology, Indiana UniversityBloomington, IN, USA; ^2^Department of Biology, Miami UniversityOxford, OH, USA

**Keywords:** individual based models, microbial diversity, dormany, encounter rate, scaling, energy limitation, deep biosphere, seed bank

## Abstract

Microbial dormancy leads to the emergence of seed banks in environmental, engineered, and host-associated ecosystems. These seed banks act as reservoirs of diversity that allow microbes to persist under adverse conditions, including extreme limitation of resources. While microbial seed banks may be influenced by macroscale factors, such as the supply of resources, the importance of microscale encounters between organisms and resource particles is often overlooked. We hypothesized that dimensions of spatial, trophic, and resource complexity determine rates of encounter, which in turn, drive the abundance, productivity, and size of seed banks. We tested this using >10,000 stochastic individual based models (IBMs) that simulated energetic, physiological, and ecological processes across combinations of resource, spatial, and trophic complexity. These IBMs allowed realistic dynamics and the emergence of seed banks from ecological selection on random variation in species traits. Macroscale factors like the supply and concentration of resources had little effect on resource encounter rates. In contrast, encounter rates were strongly influenced by interactions between dispersal mode and spatial structure, and also by the recalcitrance of resources. In turn, encounter rates drove abundance, productivity, and seed bank dynamics. Time series revealed that energetically costly traits can lead to large seed banks and that recalcitrant resources can lead to greater stability through the formation of seed banks and the slow consumption of resources. Our findings suggest that microbial seed banks emerge from microscale dimensions of ecological complexity and their influence on resource limitation and energetic costs.

## Introduction

Most microorganisms live in environments where they experience energy limitation, resource limitation, or both (Hoehler and Jørgensen, 2013; Moore et al., 2013). Microorganisms have evolved an expansive repertoire of traits that allow them to persist under conditions of resource scarcity (Follows and Dutkiewicz, 2011; Lever et al., 2015; Litchman et al., 2015). One strategy that is important for microorganisms that experience resource limitation is dormancy, i.e., a reversible state of reduced metabolic activity (Jones and Lennon, 2010; Aanderud et al., 2016). Dormant microorganisms make up a seed bank, which contributes to the maintenance of diversity (Lennon and Jones, 2011; Aanderud et al., 2015) and the functioning of ecosystems (Wang et al., 2015). Transitions into and out of dormancy are often driven by the availability of energy and nutrients (Lennon and Jones, 2011); yet seed banks still accumulate in otherwise resource-rich environments. For example, >90 % of microbial biomass in soils can be dormant even though organic matter content in these habitats can be quite high (Alvarez et al., 1998; Lennon and Jones, 2011; Blagodatskaya and Kuzyakov, 2013). Therefore, seed-bank dynamics may be influenced by factors other than macroscale properties such as the bulk concentration or supply rate of resources.

In an idealized system with few trophic interactions and where labile substrates are homogenously distributed, encounter rates between individual microorganisms and resources are governed by physical processes, such as turbulence and diffusion (Dusenbery, 2009; Rusconi and Stocker, 2015). However, these idealized well-mixed conditions are rarely met in nature. Instead, microorganisms live in complex habitats where aggregated particles of many resource types can vary in size, energetic yield, and spatial distribution (e.g., Hernández and Hobbie, 2010; Macalady et al., 2013). Such complexities modify the rate at which microorganisms encounter consumable resource particles (Kiørboe et al., 2002; Andersen et al., 2016; Großkopf and Soyer, 2016). Because it is challenging to integrate this fine-scale complexity into empirical studies, microorganisms are often investigated at spatial scales that exceed the scales of their individual interactions (Fierer and Lennon, 2011; Vos et al., 2013). For this reason, microorganisms may be nutrient- or energy-limited even though macroscale measurements would suggest that their habitat is replete with resources (Don et al., 2013; Allison et al., 2014). This phenomenon has led to the hypothesis that there is an advantage to maintaining large but inactive populations (i.e., seed banks) that are able to maximize the probability of encountering resources that vary in time or space (Vaqué et al., 1989).

The development of microbial seed banks may be influenced in part by microbe-resource encounter rates. These encounter rates are likely driven by interacting dimensions of ecological complexity. For example, microorganisms have highly aggregated spatial distributions in physically structured habitats (Raynaud and Nunan, 2014) but also in seemingly well-mixed systems (Azam and Malfatti, 2007). Such patterns may reflect the non-random distribution of resources and the capacity of microorganisms to disperse (Mitchell and Kogure, 2006; Smriga et al., 2016). Encounter rates may also be affected by the resource pool, which often comprises diverse substrates with complex molecular structures (Muscarella et al., 2014; Logue et al., 2016). Some of these resources may only be accessible to specialized taxa that produce extracellular enzymes (Lennon, 2007), which require energy that could otherwise be used for maintenance and growth (Traving et al., 2015). Last, encounters between microorganisms and resources may be influenced by trophic interactions, such as competition, predation, and parasitism (e.g., Hibbing et al., 2010). Resource-limited microorganisms also engage in the consumption of dead cells (i.e., scavenging) and the division of labor that arises from the sharing of metabolites with neighboring cells (i.e., cross-feeding) (Rozen et al., 2009; Pande et al., 2015). Together, varying degrees of spatial, trophic, and resource complexity should influence encounter rates in ways that drive growth, abundance, and activity of microbial communities, in part, through the emergence of seed banks.

Studying complex interactions at the microscale is a profound challenge for microbial ecology (Cordero and Datta, 2016). Individual-based models (IBMs) provide a way to address this challenge by exploring how individual-level interactions give rise to higher-order phenomena at the scale of populations, communities, and ecosystems (Grimm et al., 2005; Hellweger et al., 2016). IBMs can include degrees of realism and complexity that are unattainable with other types of modeling, and which lead to the emergence of unexpected but insightful phenomena (DeAngelis and Gross, 1992; Grimm, 1999; Grimm et al., 2005; Rosindell et al., 2015). In this study, we developed an IBM framework that explicitly simulates the physiology, life history, energetics, and metabolic activity of microorganisms while exploring dimensions of resource, spatial, and trophic complexity. Using randomized model parameters and random combinations of species traits, and then iterating stochastic life history processes over thousands of generations of ecological selection, we expected microbial seed banks and dynamics of growth and abundance to emerge from individual-level interactions. We show how microscale aspects of resource, spatial, and trophic complexity can drive encounter rates which, in turn, influence aspects of microbial community structure, including abundance, productivity, and seed bank dynamics. Finally, our models provide evidence of the functions that seed banks perform in energy- and nutrient-limited environments.

## Methods

### Overview of individual-based modeling

We constructed an automated source-code that built and ran stochastic individual-based models (IBMs) starting with random values of resource supply and species traits as well as random combinations of spatial, resource, and trophic complexity. These IBMs simulated physical encounters between organisms and resource particles of realistic size within spatially explicit three-dimensional environments. The purpose of this computationally demanding approach was to examine how spatial, resource, and trophic complexity affect cell-resource encounter rates and how encounter rates influence the abundance, productivity, and activity of microbial communities. In the following sections we describe how the models were parameterized with random species-, resource-, and individual-level parameters, and how the models simulated (1) realistic microscales of space and time, (2) organisms, species, and resource particles, (3) life history processes and their energetic costs as well as encounter-limited growth, and (4) levels of spatial complexity, resource complexity, and trophic complexity.

### Randomized model parameterization

The parameter values of each IBM were randomly chosen to fulfill several requirements (Table 1). These parameters included upper limits on energetic constraints (e.g., maintenance energy) and species vital rates (e.g., dispersal, growth), and also included resource-related variables (e.g., inflow rate, inflowing resource diversity), as well as random combinations of spatial, trophic, and resource complexity (Table 1). Once assembled, each IBM was populated with 100 individuals whose species identities, vital rates, resource use, and maintenance energies were drawn at random (Table 1).

**Table 1:** **Parameter values for individual based models (IBMs)**.

**Parameter**	**Definition**	**Value(s)**
**ENVIRONMENTAL**		
Width, height, length	Spatial extent of the three-dimensional environment	43,200 μm
**RESOURCE-RELATED**		
Resource supply rate	Probability of a resource particle entering per time step	0.1–1.0
Resource particle size	Simulated diameter based on the equation for the volume of a sphere and realistic resource particle sizes	4000–8600 μm
Necromass value	Nutritional worth of necromass	1–100
Resource diversity	Number of resource types that are supplied	1–10
Resource-specific “lock-and-key” constraint	For “lock-and-key” models, the probability of breaking down a resource particle in a given time step	0.01–1.0
**SPECIES TRAITS**		
Specific maintenance	Value deducted from an individual's cell quota for maintenance. Intended to be independent of cell size	0.001–0.01
Dispersal rate	Greatest distance individuals can disperse in one time step, a percent of 43,200 μm	1–100%
Resuscitation rate	Probability that each individual has of randomly resuscitating; varies among species	0.001–0.01
Maintenance-reduction	Amount by which maintenance energy is decreased when transitioning to dormancy; varies among species	10–100%
Immigration rate	Probability of an individual entering per time step	0.01–0.1
Growth rate	Species-specific probability of reproducing per time step	0.1–1.0
Log-series parameter (α)	Species form of the log-series distribution, often used to simulate ecological metacommunities, which generally takes values greater than 0.95	0.95–0.99

*Values for input parameters were randomly chosen with ranges that fulfilled several requirements, including: (1) spanning an order of magnitude, (2) biologically reasonable values when available, (3) ranges that produced computationally feasible abundances of organisms and resource particles (up to ~10^4^) within reasonable simulation times (up to several minutes for a single IBM). Each time step represented 20 min of real time, i.e., the amount of time a cell of E. coli can either reproduce once or disperse across 43,200 μm*.

Our extensive randomization accomplished several objectives. First, the IBMs were able to generate diverse trait combinations and combinations of ecological complexity across thousands of models. Second, the IBMs were able to simulate realistic but variable capacities for dormancy and realistic relationships between rates of reproduction and rates of dispersal. Third, the ranges of model parameters naturally limited organism abundance and the total number of resource particles to less than 10,000, which was necessary for practical computational overhead.

In addition to allowing traits, trade-offs, and life-history strategies to emerge as a result of ecological selection operating over thousands of generations, our randomized parameterization and stochastic simulation addressed a critical challenge to ecological modeling. Specifically, when traits, trade-offs, or life-history strategies are enforced within a model, they cannot provide evidence of the forces that produce them. However, the emergence of traits, trade-offs, and life-history strategies from initially random conditions within our models would provide evidence about the importance of microscale factors in driving rates of encounter between individual organisms and resources and, in turn, the importance of microbe-resource encounter rates in driving abundance, productivity, and the emergence of seed banks.

### Spatial and temporal scale

We chose spatial and temporal scales that were relevant to individual microorganisms, but which also established a space-time equivalency between rates of reproduction and dispersal. For example, one of the most well-studied microorganisms (*Escherichia coli*) is believed to disperse up to 36 microns (μm) per second (Milo and Phillips, 2015). Likewise, under ideal conditions, *E. coli* is able to double every 20 min (Wang et al., 2010). Using 1200 seconds (20 min) as a minimum doubling time (i.e., nothing doubling faster than *E. coli*) and 36 μm per second as a maximum specific dispersal rate, our models obeyed a space-time equivalency such that each time step allowed, at most, one doubling per individual for the fastest reproducing species or 36 μm of dispersal for the most quickly dispersing species. We then constrained each time step to 0.12 s, translating to 1200 simulated real-world seconds and, hence, a three-dimensional spatial extent of 43,200 μm in each direction for a total volume of 8·10^4^ cm^3^. For context, densities of bacteria can reach several billion per cm^3^ in resource-rich environments, such as soils, sediments, and gut contents (Whitman et al., 1998). In our modeling, we aimed to simulate at most a few tens of thousands of individual organisms and resource particles, primarily due to computational limits but also to simulate sparsely populated environments that were highly limited in energy-yielding resources.

### Simulating individuals, species, and resources

Our modeling simulated different types of resource particles that could be consumed by heterotrophic microorganisms belonging to different species. We used data objects (e.g., lists and dictionaries) that are native to most computing languages. Individuals were distinguished by collections of elements within dictionaries, which are data objects that use key-value pairs to assign values (e.g., growth rate) to a “key” (e.g., individual ID). For example, a dictionary of information for individual “1” could be as follows: {“quota”: 0.5, “x”: 50, “y”: 100}. Here, “quota” refers to the individual's cell quota, i.e., amount of endogenous resource. This individual would have half of its maximum cell quota (i.e., 0.5), and would be located at the x-y coordinates of 50–100. Each individual and its dictionary of information was then held in a community-level dictionary (INDs):

$$\begin{array}{l} \text{INDs}=\{1:\{\text{species}:12,\text{quota}:0.51,\text{x}:5320,\text{y}:10,012,\text{z}:\\ \;41,991\},\\ \;\{2:\{\text{species}:7,\text{quota}:0.10,\text{x}:20,564,\text{y}:8822,\text{z}:\\ \;687\},...\} \end{array}$$

Species-specific information including specific rates of growth and dispersal, values of cellular maintenance, probability of randomly reactivating, resource specificity, etc., were held in a community-level species dictionary (SPs):

$$\begin{array}{l} \text{SPs}=\{12:\{\text{growth}:\text{0}.\text{11},\text{dispersal}:\text{0}.\text{45},\text{maintenance}:\text{0}.\text{01}\},\\ \;\{7:\{\text{growth}:\text{0}.\text{25},\text{dispersal}:\text{0}.\text{02},\text{maintenance}:\\ \;0.0015\},...\} \end{array}$$

In simulating an ecological process (e.g., growth, dispersal, cell maintenance) a model would choose an individual (e.g., 1, 2, …) at random and then use the information for the individual in INDs and its species to determine how much the individual grows when it encounters a resource, how fast it disperses, how much cell quota is lost to maintenance, etc. Like individuals, resource particles were distinguished by collections of elements within dictionaries. When resource particles entered the system, they were assigned a resource identity (e.g., a, b, c) at random, a value between 1000 and 10,000 representing the size of the resource particle, as well as a set of three-dimensional spatial coordinates. Each particle was also given a unique identity (ID) for referencing the resource dictionary.

$$\begin{array}{l} \text{RESs}=\{1:\{\text{type}:\text{b},\text{size}:\text{10000},\text{x}:\text{1520},\text{y}:\text{55},\text{104},\text{z}:\text{370}\},\\ \;\{2:\{\text{type}:\text{a},\text{size}:\text{1500},\text{x}:\text{25},\text{001},\text{y}:\text{18},\text{150},\text{z}:\\ \;5450\},...\} \end{array}$$

More information on the simulation of individual organisms, resource particles, and all other aspects our modeling can be found in a highly detailed version of the standard IBM protocol of Grimm et al. (2006), which is available as a supplemental file (see Supplemental File 1) and in a public GitHub repository (https://github.com/LennonLab/Micro-Encounter).

### Simulating life history processes

Our IBMs used uniform random sampling to choose which individuals at a particular moment would undergo specific life history processes, such as growth, reproduction, death, cellular maintenance, dispersal, and transitions into an out of dormancy. Whether a randomly chosen individual underwent a specific life history process was determined, in part, by the individual's cell quota, species-specific trait values, and distance to resource particles. Modeling in this probabilistic way simulated the partly probabilistic and partly deterministic nature of environmental filtering and individual-level interactions. The order in which processes occurred was allowed to vary at random from time step to time step, which prevented artifacts from arising that might be driven by the ordering of simulated life history processes. Specifically, we implemented life history process as follows:

#### Immigration

Individuals entered the system at any point in the three-dimensional environment. Species identities of inflowing propagules were chosen at random from a uniform distribution. Individuals were assigned a unique ID and a randomly chosen cell quota between 0.5 and 1.0, which translated to starting cell diameters of 1 to 1.25 μm.

#### Consumption, growth, and cell size

Consumption increased an individual's cell quota and decreased the size of the resource that an individual had encountered. Growth was simulated as an increase in cell quota that corresponded to a proportional increase in biomass. That is, we assumed that cell quota (*Q*) and cell volume (*V*) were proportional and hence, that *Q* was related to a cell's radius through a geometric relationship: *Q* ∝ *V* = ^4^/_3_ π *r*^3^. Estimating cell size was important as the ability to consume a resource particle relied on whether a cell and a resource particle were in physical contact. The energetic cost of growth was proportional to the product of growth rate and individual cell quota, such that larger individuals required more energy to increase by a fraction of their body mass.

#### Physiological maintenance

Individual cell quotas diminished according to a randomly chosen species-specific maintenance cost, i.e., a numeric value subtracted from the cell quota. This cost was the implied energetic cost of maintaining basal metabolism and cellular maintenance. When active individuals transitioned to a dormant state, the cell maintenance cost decreased by a species-specific factor that was likewise chosen at random. This cell maintenance cost was reversed when individuals resuscitated to a metabolically active state.

#### Reproduction

Reproduction was clonal and occurred via binary fission and without mutation. Individuals reproduced with a probability directly determined by the ratio of cell quota to maximum cell quota (i.e., 1000). Hence, the probability of reproducing when randomly sampled was between 0.0 and 1.0. The daughter cell was given a unique individual ID along with the species ID and geographical coordinates of its mother cell.

#### Death

An individual sampled at random died if its cell quota was less than the species-specific maintenance energy, whether in an active or dormant state. Depending on the model, the remains of dead individuals could be scavenged and consumed by other cells. Otherwise, dead individuals were effectively lost to the system, as in most community ecology models.

### Simulating resource breakdown and consumption

Resource particles entered the system with diameters ranging between 5000 and 20,000 μm. When encountered, individuals would consume the resource according to species specific rates of consumption. The remaining portion of the resource particle would then be randomly broken in two parts. Below, we describe how, depending on the model, the specific details of resource breakdown and consumption varied with regard to whether the model included scavenging, cross-feeding, or resources of varying complexity or recalcitrance, i.e., the difficulty of being broken down.

### Simulating ecological complexity

**Figure 1:**
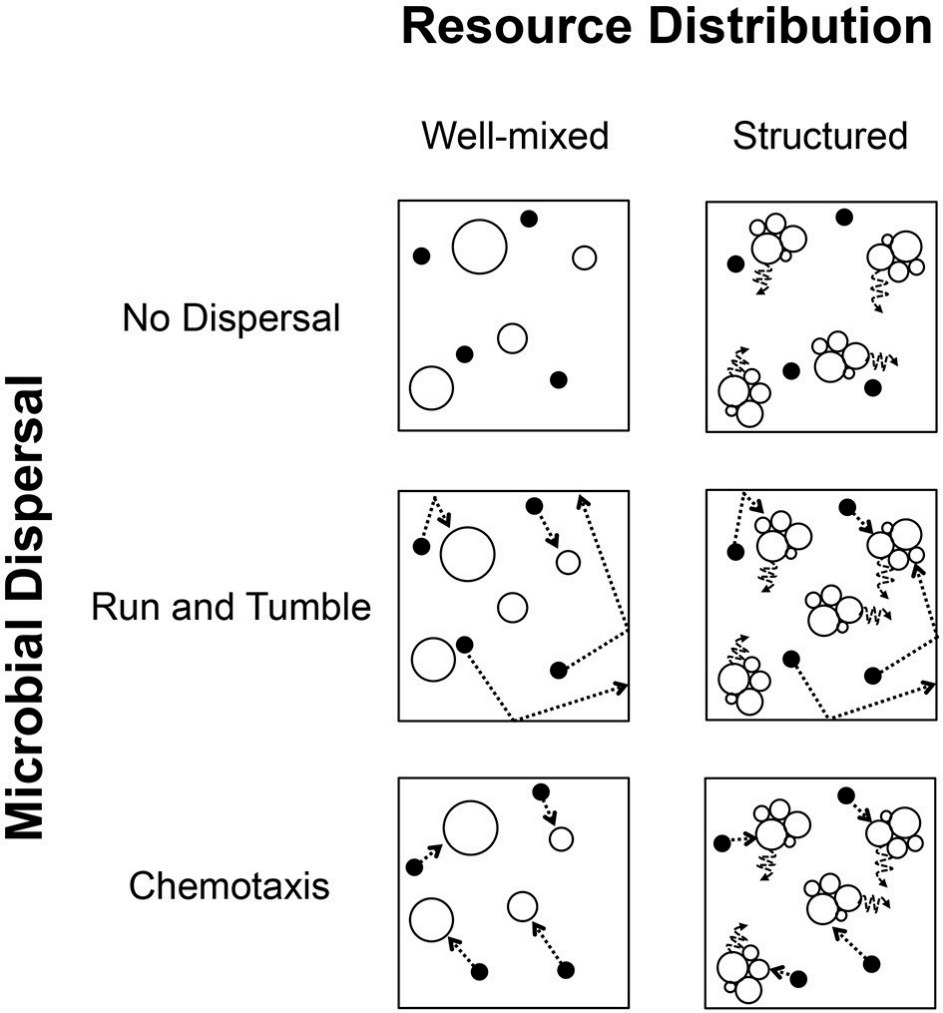
**Conceptual diagram depicting conditions of spatial complexity simulated in individual based models (IBMs)**. Columns: The spatial distribution of resources and organisms was modeled in two ways. **(Left)** Locations of resource particles and individual organisms changed randomly between time steps, resulting in a well-mixed model of uncorrelated changes in position. **(Right)** Aggregated resource particles remained largely fixed in position and only made slight movement under Brownian motion, resulting in a highly structured and spatially heterogeneous environment. Rows: Whether in a well-mixed or structured environment, individuals could change position under three modes of dispersal. **(Top)** Individuals could disperse passively (no active dispersal), relying entirely on either the well-mixed conditions or Brownian motion imposed by their environment. **(Center)** Individuals could actively disperse along a trajectory and change direction upon hitting a barrier or edge, in a “run and tumble” fashion. **(Bottom)** Most energetically expensive, individuals could actively disperse in a directed manner after sensing the presence of consumable resources, i.e., chemotaxis. In the modeling, each condition of resource distribution was compatible with all conditions of individual dispersal, resulting in 6 possible combinations.

**Figure 2:**
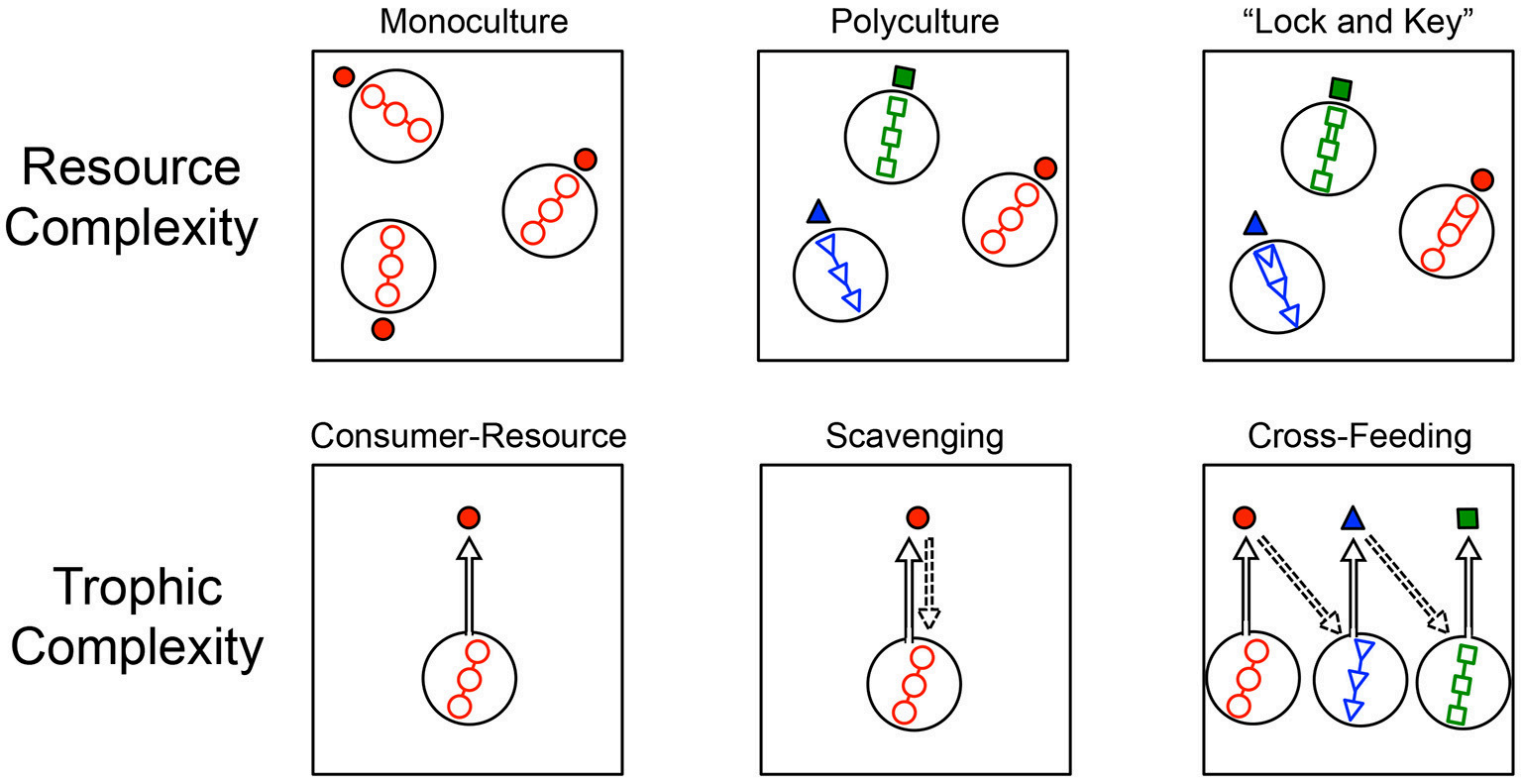
**Conceptual diagram depicting aspects of resource complexity and trophic complexity simulated in individual based models (IBMs)**. Resource complexity **(top row)** was simulated in three ways. **(Left)** All resources supplied from outside the system to an individual microbe (solid red circle) were of the same type (open red circles), resulting in monoculture conditions. **(Center)** All resources supplied from outside the system to microbial taxa (solid red circles, solid blue triangles, solid green squares) were of three different types (open blue triangles, open green squares, open red circles) supplied in equal proportions and resulting in a polyculture. **(Right)** Resources required energetically costly efforts to breakdown and use, much like an enzymatic “lock-and-key” in the breakdown of complex macromolecules. Models were defined by either monoculture or polyculture resource conditions, and also had either “lock-and-key” resource complexity or simple resources that were immediately assimilated without an energetic cost. Trophic complexity **(bottom row)** was also simulated in three ways. **(Left)** A consumer-resource condition where all species belonged to the same trophic level and where individuals only consumed externally supplied resources. **(Center)** A scavenging condition where the remains of dead individuals were consumed by any other individual in the system that was in close enough physical proximity. **(Right)** A cross-feeding condition where individuals produced metabolites or by-products that were then consumed by individuals belonging to another taxon. In the modeling, cross-feeding could emerge as a one-way relationship (pictured) or as a two-way relationship.

In addition to explicitly simulating the spatial environment and individual-level changes in organisms and resource particles, we constructed our modeling framework to allow random combinations of various levels of spatial, resource, and trophic complexity. Each IBM was parameterized at random with one of 72 combinations of complexity (4 trophic, 3 resource, 3 dispersal, 2 spatial resource distribution) (Figures 1, 2). We then explored how these dimensions of complexity affected encounter rates, along with attributes, such as total abundance (i.e., total number of individual in a community; *N*), the abundance of active individuals, production of individuals per time step (i.e., productivity), and the relative size of the microbial seed bank (i.e., percent dormancy).

#### Spatial complexity

We simulated levels of spatial complexity in terms of the spatial structure of the environment and modes of individual dispersal (Figures 1, 2). The first level of spatial complexity in the environment was a well-mixed model in which the locations of individual organisms and resource molecules changed at each time step in an uncorrelated way. Hence, every organism and resource particle had the same chance of moving to any location within the environment at each time step in the model. While this is unrealistic in highly structured systems of relatively large size, the microscale spatial dynamics of more fluid systems can be highly complex, rapidly mixed, and unpredictable (Rusconi et al., 2014). This model created a well-mixed environment and allowed for passive dispersal. In the second level of spatial complexity, resource molecules only changed their location via Brownian motion, resulting in a system that lacked mixing and where, through the breakdown of large resource particles, smaller resource particles became aggregated. We refer to this second level as “spatially structured.”

The first mode of individual dispersal excluded active dispersal and was entirely passive. While passive dispersal has the benefit of not requiring an energetic investment, we expected this mode of dispersal to only have a benefit under well-mixed conditions. The second mode of dispersal allowed active movement, but did not allow chemoreception. This “run and tumble” strategy allowed individuals to move along a straight line in a randomly chosen direction and, once hitting the edge of the environment, turned and moved in a new, randomly chosen direction. By chance alone, individuals would be able to encounter resource particles. The third mode of dispersal simulated chemotaxis, i.e., the ability to sense the location of resources and actively move toward them. Because chemotaxis involves sense perception, we encoded it as a more energetically expensive mode of dispersal. In this way, one could envision a potential trade-off between the greater energetic cost of chemotaxis and the efficiency associated with more rapid encounter of resources.

#### Resource complexity

Our simulations captured important features of resource complexity that we hypothesized would affect encounter rates. First, we considered resource diversity, which refers to the different types of resources that are supplied to a system. In the “monoculture” simulation, only one type of resource was supplied. Second, we considered a “polyculture” scenario, where three different types of resources were supplied and each could only be used by specialist consumers (Figure 1). Third, we considered that some resource particles are more difficult to break down, or recalcitrant, than others. To simulate recalcitrance, we imposed a “lock and key” scenario that required individuals to invest time and energy to break down complex molecules for consumption. Importantly, a given model could include this “lock and key” feature while being a monoculture or polyculture.

#### Trophic complexity

We simulated three aspects of trophic complexity that we hypothesized would influence resource encounter (Figure 1). First, we simulated a simple heterotrophic “consumer-resource” interaction where all individuals were solely consumers of inflowing resources. The second level of trophic complexity allowed for “scavenging” which is specified in the model as the consumption of resources from dead individuals (e.g., Rozen et al., 2009). Our third level of trophic complexity simulated a situation wherein individuals produced metabolites as a result of breaking down and consuming resources. These metabolites could potentially be consumed by other species that, in turn, produced metabolites that also be consumed. This situation was meant to simulate conditions that are characteristic of cross-feeding or syntrophy (Pande et al., 2015). Additionally, because the metabolites that individuals of particular species produced were chosen at random at the start of the model, it was possible for mutualistic cross-feeding to occur, i.e., where two species produced metabolites that the other could consume.

### Modeling workflow

Each model was run to a state of mean reversion, i.e., a point where the total abundance of the microbial community fluctuated around a given value, as the model iterated over thousands of generations of randomly ordered life history processes. This burn-in period was then discarded and each model was run for 2000 additional generations. We recorded information for each tenth generation past the burn-in period (Table 1).

## Results and discussion

Dormancy allows microorganisms to persist in low-resource environments, yet seed banks emerge under other conditions as well (Lennon and Jones, 2011; Blagodatskaya and Kuzyakov, 2013). While it is generally assumed that nutrient and energy limitation can lead to seed banks, the interrelated variables and dimensions of ecological complexity that influence the transition of individuals into dormancy are poorly understood and are difficult to study in natural environments. Our results from >10,000 individual-based models (IBMs) suggest that microscale factors modify microbe-resource encounters in ways that influence fundamental properties of microbial communities. For example, dispersal, spatial structure, and resource complexity had a greater influence on encounter rates than macroscale properties, such as the supply rate and bulk concentration of resources in the environment. In turn, these microscale drivers served as important controls on microbial community attributes, such as abundance, productivity, and seed banks.

### Realistic model behavior

Not even the most sophisticated models can accurately simulate all factors affecting growth, abundance, and activity in complex ecological communities. Nevertheless, our IBMs reproduced several realistic features of microbial systems. First, resource particles were broken down to mean particle sizes of 3.5 to 412 μm. These ranges approximate the sizes of marine snow aggregates (Alldredge, 1998), macro- and micro-aggregates of soil (Puget et al., 2000), and suspended and sedimented detritus in marsh ecosystems (Marsh and Odum, 1979). Second, mean cell diameters ranged from 0.2 to 2.0 μm, which approximates the range of sizes from the extremely small marine bacteria SAR11 (~0.2 μm) (Rappé et al., 2002) to the relatively large diameter (0.4–0.8 μm) and length of *E. coli* (1.0–3.5 μm) (Trueba and Woldringh, 1980). Third, we found that the ecological model that has been shown to best explain patterns of commonness and rarity among microbial communities from aquatic, terrestrial, host-related systems often explained >80% of variation among species in our IBMs (Figure S1; also see Shoemaker et al., 2016). Fourth, as discussed in the following sections, we observed intuitive relationships of growth, abundance, and dormancy. These relationship reinforce reasonable model behavior and provide insights into how microscale complexity drives encounter and how encounter rates drive growth, abundance, and the emergence of microbial seed banks.

### Microscale vs. macroscale drivers of resource encounter

**Figure 3:**
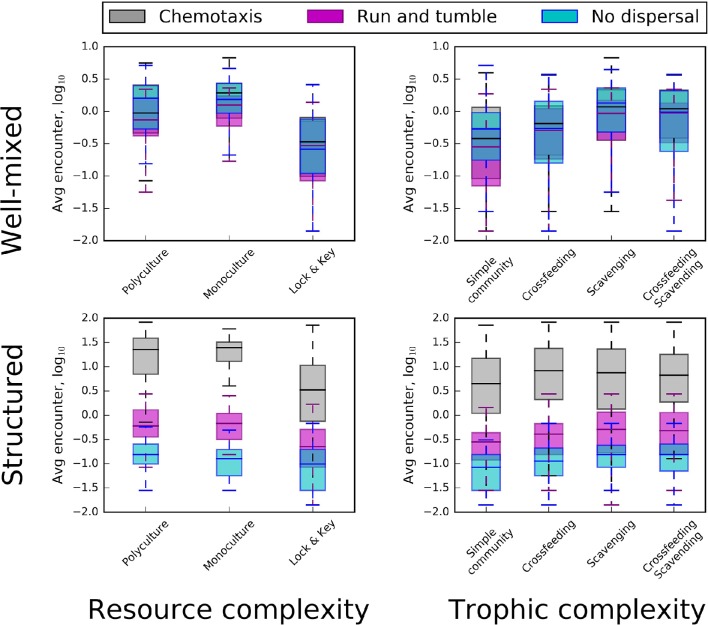
**Box plots revealing the influence resource complexity (left)**, trophic complexity **(right)**, spatial distributions **(top and bottom)**, and dispersal conditions (different colors) on frequencies of encounter between organisms and resource particles. Top row: In well-mixed environments, “lock-and-key” chemical complexity (in combination with either monoculture or polyculture conditions) had a strong influence on encounter rate, resulting in a substantial decrease. Monoculture and polyculture box plots did not include “lock-and-key” resource complexity. In the top row, (i.e., well-mixed environments) chemotaxis and “run and tumble” do not substantially change encounter rates. Bottom row: In structured environments, active dispersal overrode the influence of chemical complexity, with chemotaxis providing the greatest increase in encounter.

The consumption of resources needed for growth and reproduction are regulated by factors that influence encounter rates. While our IBMs reflected this basic assumption, we found that encounter rates were differentially influenced by macroscale and microscale properties of the simulated environment. For example, there was no relationship between encounter rate and the resource supply rate (Figure S1) and though encounter rate increased with resource concentration, the relationship was noisy and complicated (Figure S2). We also found that the diversity of inflowing resources, which ranged from 1 to 10 different types, had little-to-no influence on encounter rates (Figure 3). In contrast, several microscale properties had substantial influence on encounter rates, including dispersal mode, the recalcitrance of individual resources, and to a lesser degree, trophic complexity (i.e., scavenging, cross-feeding) (Figure 3). We discuss the nature of these microscale factors on microbe-resource encounter rates in the following subsections.

#### Active dispersal

Encounter rates were most affected by interactions between dispersal mode and the spatial structure of the environment (Figure 3). In well-mixed environments, neither chemotaxis nor “run and tumble” produced greater encounters than the energetically free strategy of passive dispersal. However, in structured environments, chemotaxis produced 10–100 times more encounters while passive dispersal frequently resulted in no encounters. Chemotaxis is an important microbial trait that has strong effects on resource encounter rates (e.g., Datta et al., 2016; Smriga et al., 2016). The energetic cost of this directed movement must have been offset by the energy saved in not encountering resources at random, as with our simulated “run and tumble” mode of dispersal. It is generally assumed that chemotaxis is a trait that is favored in productive habitats and selected against in oligotrophic environments where searching for sparse chemically complex resources could be energetically wasteful (Ottemann and Miller, 1997). This view is supported by genomic evidence suggesting that bacteria from productive environments tend to harbor more motility genes than bacteria from oligotrophic environments (Giovannoni et al., 2005; Lauro et al., 2009). Though our models did not support these observations, the energetic cost of chemotaxis, as modeled, may not have been great enough to substantially tax individual cell quotas. More likely, however, individuals that became energetically depleted through chemotaxis simply went dormant.

#### Spatial distribution

Habitat heterogeneity is thought to limit the probability of decomposition and to influence the accumulation of resources in spatially structured environments like soil (Schmidt et al., 2011; Dungait et al., 2012; Lehmann and Kleber, 2015). Our simulations support this view. Except in the case of chemotaxis, we found that encounter rates were lower in spatially structured environments, which resulted in the aggregation and persistence of resources in the environment (Figure 3). This pattern is most clearly seen in the comparisons between mixed and structured environments, where the “run and tumble” strategy or passive dispersal resulted in greatly reduced encounter rates (Figure 3). Our results highlight the benefits of chemotaxis in overcoming the challenges of resource acquisition in spatially structured environments. On the other hand, in well-mixed environments, chemotaxis did not result in greater encounters compared to the energetically less expensive “run and tumble” mode of dispersal and the energetically free passive dispersal strategy. These model-based findings suggest the importance of considering energetic trade-offs related to modes of dispersal and the spatial distribution of resources.

#### Resource complexity

**Figure 4:**
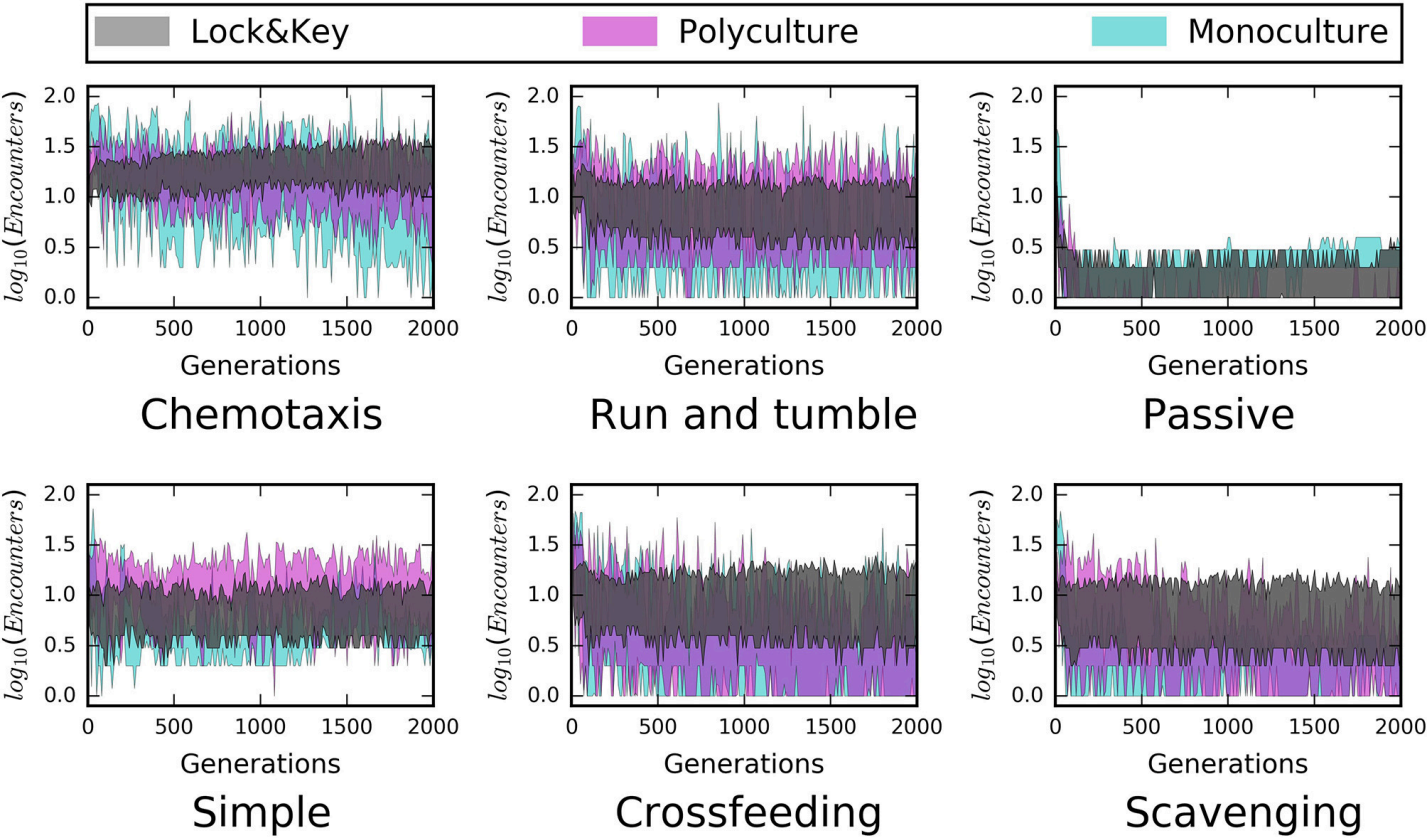
**Time series from structured environments reveal a stabilizing influence of “lock-and-key” resource chemical complexity on microbe-resource encounters**. Both “monoculture” and “polyculture” hulls (75% confidence intervals) pertain to simple (i.e., labile) resources, and have higher variability in encounter rates than the “lock-and-key” models. This was the case across all modes of dispersal and all modeled forms of trophic complexity (i.e., simple consumer-resource model, cross-feeding, scavenging).

The recalcitrance of resources reduced encounter rates in both mixed and structured environments (Figure 3). In order for microorganisms to consume complex resources, they needed to invest energy in cleaving resource particles into consumable parts, slowing encounter with those breakdown products. This finding confirms how structurally complex or recalcitrant resources are thought to influence the growth and activity of microorganisms within habitats, such as soil (Schimel and Weintraub, 2003; Allison et al., 2011). Along with the spatial distribution of resources, chemical complexity slows the loss of energy from the resource pool by limiting encounter. This “slow release” effect has been hypothesized to affect ecosystem dynamics (e.g., Wetzel, 1999) and its interaction with spatial structure is consistent with emerging views on the controls of organic matter persistence in soil environments (Lehmann and Kleber, 2015). Furthermore, time series of our simulated data suggest that the slow turnover of recalcitrant substrates contributes to a more stable dynamic of resource encounter (Figure 4). Whereas the inclusion of complex resources (i.e., “lock-and-key” models) had little-to-no influence on average encounter in well-mixed models, the presence of recalcitrant resources did impart greater stability in encounter rates over time, regardless of dispersal mode or trophic complexity (Figure 4).

#### Trophic complexity

Our simulations revealed that trophic complexity had only modest effects on encounter rates (Figure 3). Both scavenging and cross-feeding led to slight increases in resource encounter in both well-mixed and structured environments across all modes of dispersal. However, the small effect of trophic complexity on resource encounter should be cautiously interpreted. First, we only considered a few types of trophic interactions and we encoded them in fairly specific forms. In addition to scavenging and cross-feeding, microbial communities engage in a plethora of trophic interactions, which could affect encounter and consumer-resource interaction strength. Second, aspects of our modeling may have dampened the effects of scavenging and cross-feeding on encounter rates. For example, because our models included dormancy which reduces mortality, seed banks may have reduced the importance of scavenging by decreasing available necromass. Finally, it is possible that scavenging and cross-feeding affected resource encounter for a small cross-section of models but that the signal was overshadowed by the larger effects of spatial and resource complexity. We envision two ways that future studies could more closely examine the roles of trophic complexity in driving microbe-resource encounters. First, studies could explore the influence of trophic levels or ask whether trophic interactions that lower the abundances of primary consumers (e.g., predator-prey, parasitism) drive resource encounters. Second, the possibility that scavenging could increase the availability of resources to organisms warrants greater attention to the energetic value derived from necromass.

### Influence of encounter on microbial dynamics

**Figure 5:**
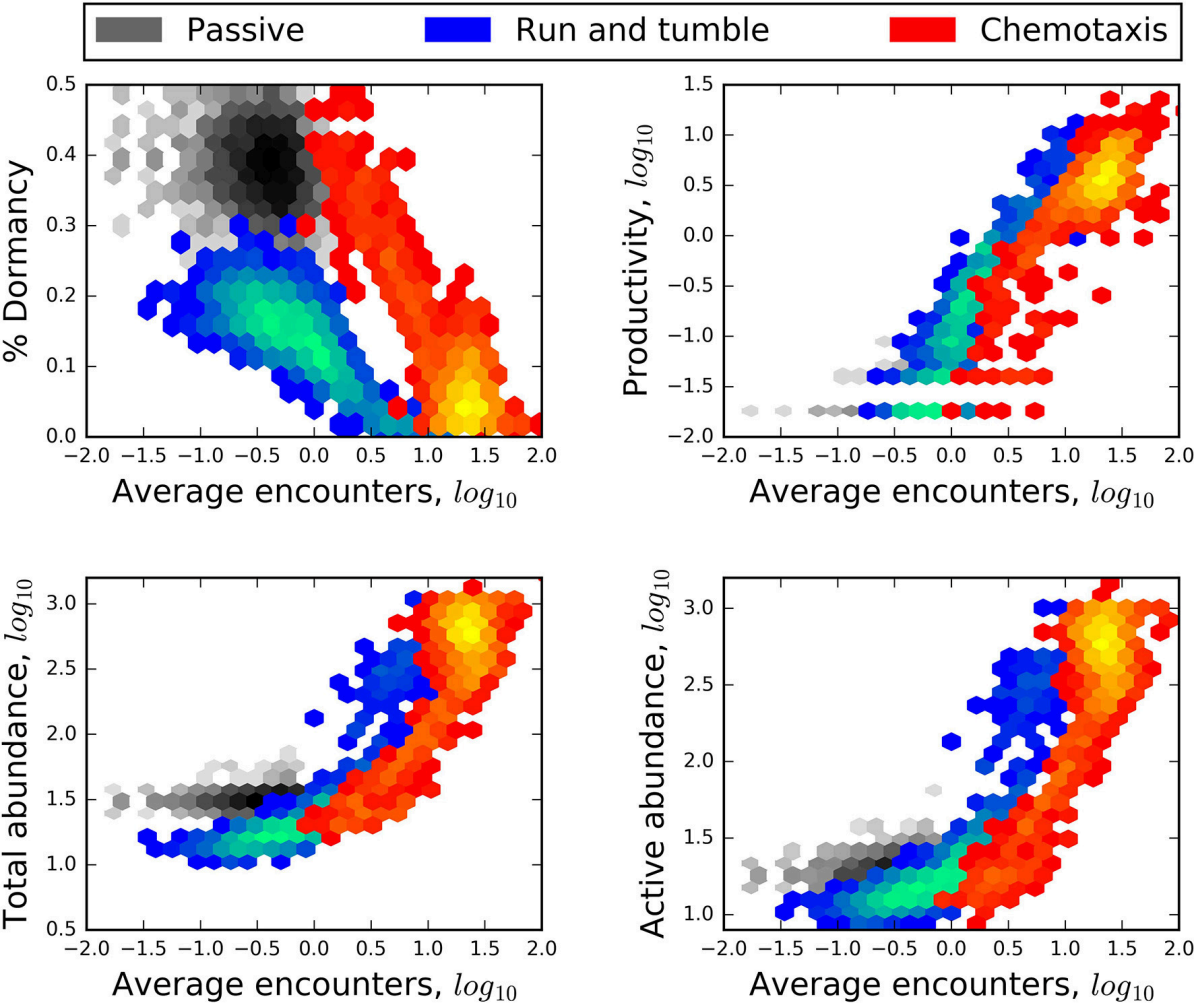
**Heat maps revealing a negative relationship of encounter to the relative size of the seed bank (% dormancy) and positive relationships of encounter to the production of new individuals (productivity), and to the abundance of the total and active communities**. In each plot, blue heat maps represent the results of models that included “lock-and-key” chemical complexity (complex molecules) and excluded active dispersal. Red heat maps represent the results of models that excluded chemical complexity (simple molecules) but included chemotaxis. Greater heat, i.e., areas within heat maps that have lighter colors, corresponds to a greater number of model results.

Aspects of abundance, productivity, and activity were strongly related to encounter rates (Figure 5). We found that microbial seed banks emerged from multiple mechanisms related to resource-mediated energetic limitations. Finally, both microbial seed banks and a slow release effect of recalcitrant resources may stabilize microbial systems against large fluctuations in growth and resource availability.

#### Abundance, productivity, and emergence of seed banks

Greater resource encounters led to greater productivity and greater total abundance, and smaller seed banks (Figure 5). While these relationships can be expected since resource encounters are required to fuel growth, we also observed that modes of dispersal influenced the general forms of these relationships. For example, in spatially structured models, chemotaxis not only led to greater encounters but also some of the largest seed banks. In contrast, the “run and tumble” strategy produced the overall smallest seed banks despite encounter rates that were often very low (Figure 5). We attribute the stark difference between these two strategies to their energetic cost, where chemotaxis was twice as energetically costly as “run and tumble”. Resource complexity and trophic complexity had no effect on relationships of abundance and productivity to rates of encounter regardless of whether the environment was structured or well-mixed.

**Figure 6:**
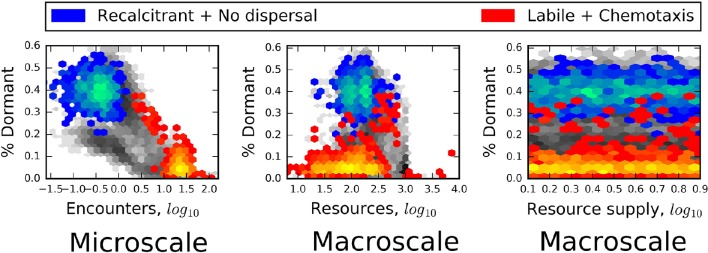
**Heat maps reveal that the relative size of the seed bank (% Dormant) was strongly related to the microscale property of encounter but was independent of the macroscale properties of total resources and resource supply**. In each plot, blue heat maps represent the results of models that included chemical complexity (recalcitrant; complex molecules) and excluded active dispersal. Red heat maps are results of models that excluded chemical complexity (labile; simple molecules) but included chemotaxis. Greater heat, i.e., areas within heat maps that have lighter colors, corresponds to a greater number of model results.

**Figure 7:**
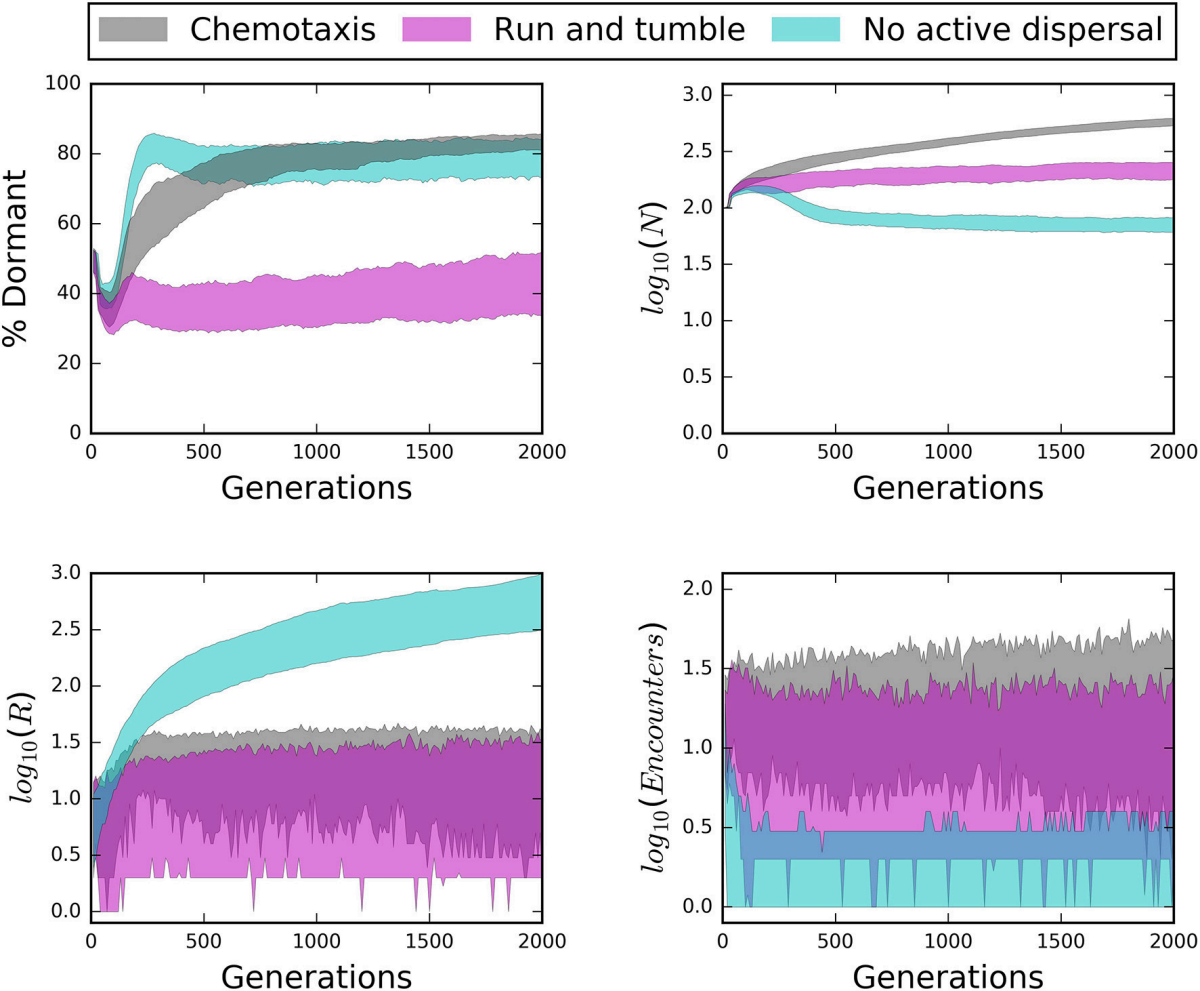
**Time series of structured environments reveal the influence of dispersal mode (i.e., chemotaxis, “run and tumble,” no active dispersal) on seed bank size (% Dormant), total abundance (*N*), total resources (*R*), and the number of encounters**. Chemotaxis generally produced the largest seed banks while “run and tumble” produced the smallest seed banks, even though chemotaxis resulted in the greatest encounters; a potential consequence of the energetic cost of chemotaxis. Under passive dispersal, the community was largely dormant, small in abundance, and experienced low encounters even as resource particles accumulated.

Consistent with energy- and nutrient limitation as a control on microbial activity, we observed that seed banks were largest in simulations that included recalcitrant resources and passive dispersal (Figure 6). In contrast, the relative size of seed banks was not related to the macroscale properties of total resource supply and bulk resource concentration (Figure 6). Finally, we often found that passively dispersing individuals did not encounter resource particles (Figure 7). On closer inspection, this appeared to be due to the small size and sparse spatial distribution of many resource particles, i.e., “crumbs,” which has been suggested as an alternative to resource recalcitrance as a reason for why large amounts of total resources seem to be resistant to microbial consumption (Arrieta et al., 2015). In soil systems, low concentrations of organic matter have been found to limit its use by microbes (Don et al., 2013), presumably because dilute resources limit the encounter with organisms. Consequently, it may be that natural systems in which we find large seed banks are likely to be those characterized by recalcitrant or chemically complex resources, such as plant structural materials, as well as sparse distributions of relatively small resource particles.

#### Influence of seed banks on temporal dynamics

**Figure 8:**
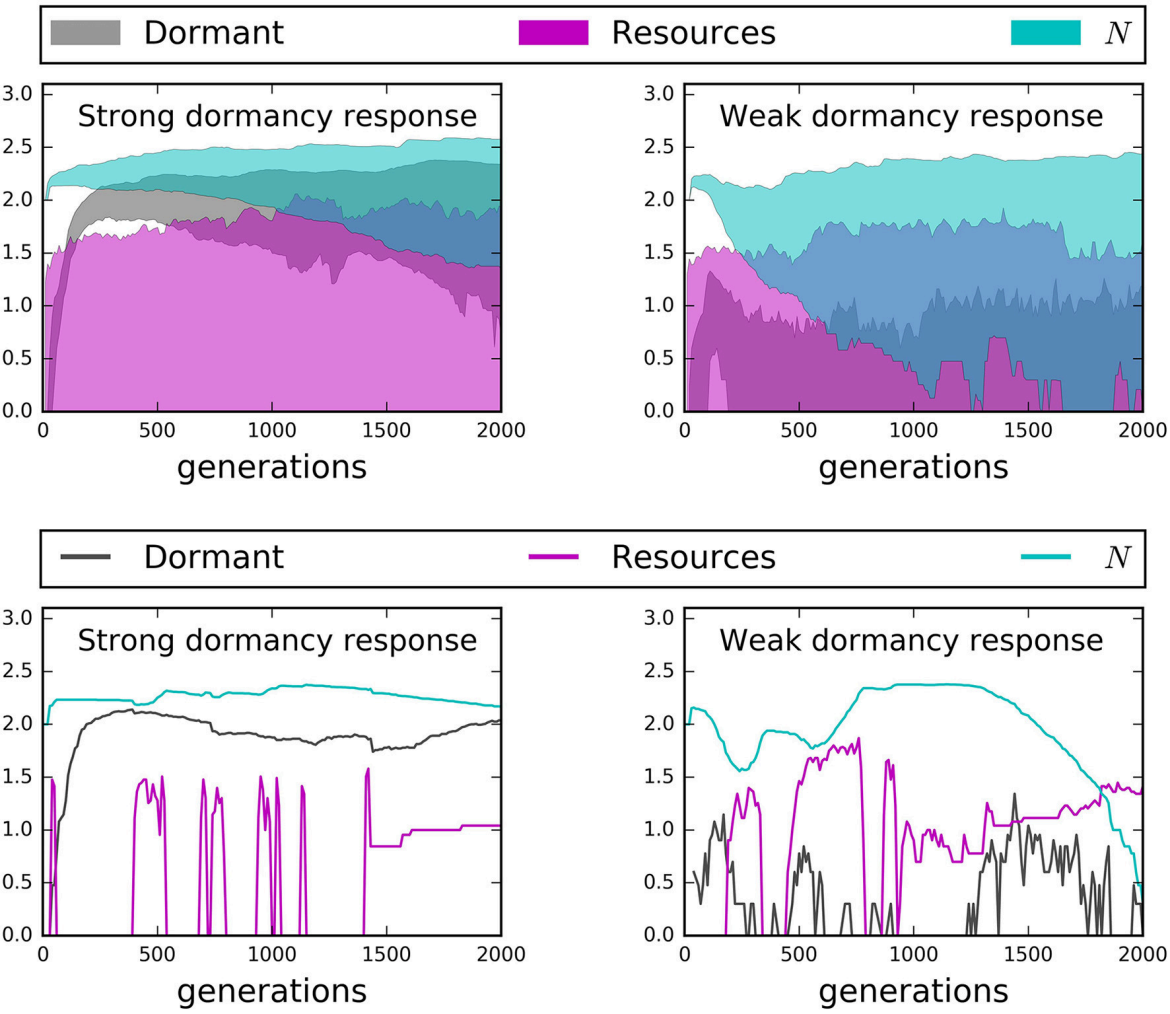
**Time series results from models with a strong dormancy response (dormancy decreases maintenance energy by a factor of 100, probability of random resuscitation = 0.001) and a weak dormancy response (dormancy decreases maintenance energy by a factor of 10, probability of random resuscitation = 0.1)**. Top row: 95% confidence hulls reveal that a strong dormancy response almost always prevents extinction while a weak dormancy response leads to greater activity but not necessarily greater total abundance (*N*). Bottom row: A random selection of two models. **(Left)** A strong dormancy response leads to a more stable *N*, despite high variability in or absence of total resources (*R*). **(Right)** A weak dormancy response can lead to extinction, even in the presence of increasing resources.

Time series from our IBMs revealed important influences of microbial seed banks across 2000 generations. Active dispersal and a strong capacity for dormancy helped maintain large and stable communities over time (Figures 7, 8). However, while chemotaxis resulted in the highest rates of encounter and greatest abundances, it also produced substantially larger seed banks than models that incorporated the less energetically expensive “run and tumble” strategy (Figure 7). This result suggests that while chemotaxis may be effective for locating resources, its energetic demands can cause organisms to enter dormancy as a consequence of exhausting their cell quotas in pursuit of resources. We found that communities went extinct less often and fluctuated less wildly when there was a high capacity for dormancy, i.e., low maintenance energy and low probability of randomly resuscitating (Figure 8). Under a weak capacity for dormancy, communities of active and passive dispersers often went extinct even when the bulk concentration of resources was high and relatively stable (Figure 8). These findings not only confirm our support the importance of seed banks to microbial communities in a variety of systems, but also indicate that extinction can occur under relatively high resource concentrations, i.e., if individuals fail to encounter and consume resources.

## Conclusion

Our study demonstrates that microbial seed banks can emerge from microscale factors that influence the accessibility of resources imposed by the spatial structure of the environment, energetic trade-offs among modes of dispersal, the structural complexity of resources, and to a lesser degree, cross-feeding and scavenging. Our study also illustrates that macroscale factors like the bulk supply and concentration of resources do not always influence encounter rates nor the emergence of microbial seed banks. These findings help explain the presence of large microbial seed banks throughout environmental, engineered, and host-associated ecosystems of high resource concentrations and supply. Likewise, our findings show that while resource limitation may be a primary driver of microbial seed banks, these limitations can drive the emergence of seed banks via multiple mechanisms. These mechanisms include the slow and consistent release of energy from resource that are not highly labile, the sparse spatial distribution of dilute resource particles, the energetic costs of foraging, and the spatial mixing of the environment. To better understand the role of microscale mechanisms in microbial systems and the importance of individual-level interactions and the role of the local environment, we suggest that ecologists pursue microscale work through the intersection of modeling and empirical studies. These efforts may require single-cell metabolic analyses of microscale samples and continued refinement of individual-based models for microbial ecology.

## Author contributions

KL, MF, and JTL designed the study. KL created models and performed analyses. KL, MF, and JTL wrote the manuscript.

### Conflict of interest statement

The authors declare that the research was conducted in the absence of any commercial or financial relationships that could be construed as a potential conflict of interest.
