# Supplementary material for: Microscale Insight into Microbial Seed Banks
Source: Front Microbiol. 2017 Jan 9;7:2040. doi: 10.3389/fmicb.2016.02040 (PMC5220057; doi:10.3389/fmicb.2016.02040)

Figure captions

FIGURE S1 | Fits of the two most popular species-abundance models, i.e., Poisson lognormal (PLN) and the log-series distribution predicted by the Maximum Entropy Theory of Ecology (METE) to simulated community data (i.e., vectors of species abundances) from >10,000 individual-based models (IBMs). Kernel density curves for the portion of variation (r^2) in species abundances explained by the PLN and METE reveal that the PLN often explained upwards 90% of variation in abundance among species across IBMs; a finding that is consistent with a recent empirical study using the largest ever compilation of microbial community data (see Shoemaker et al. 2016; https://peerj.com/preprints/1450/).

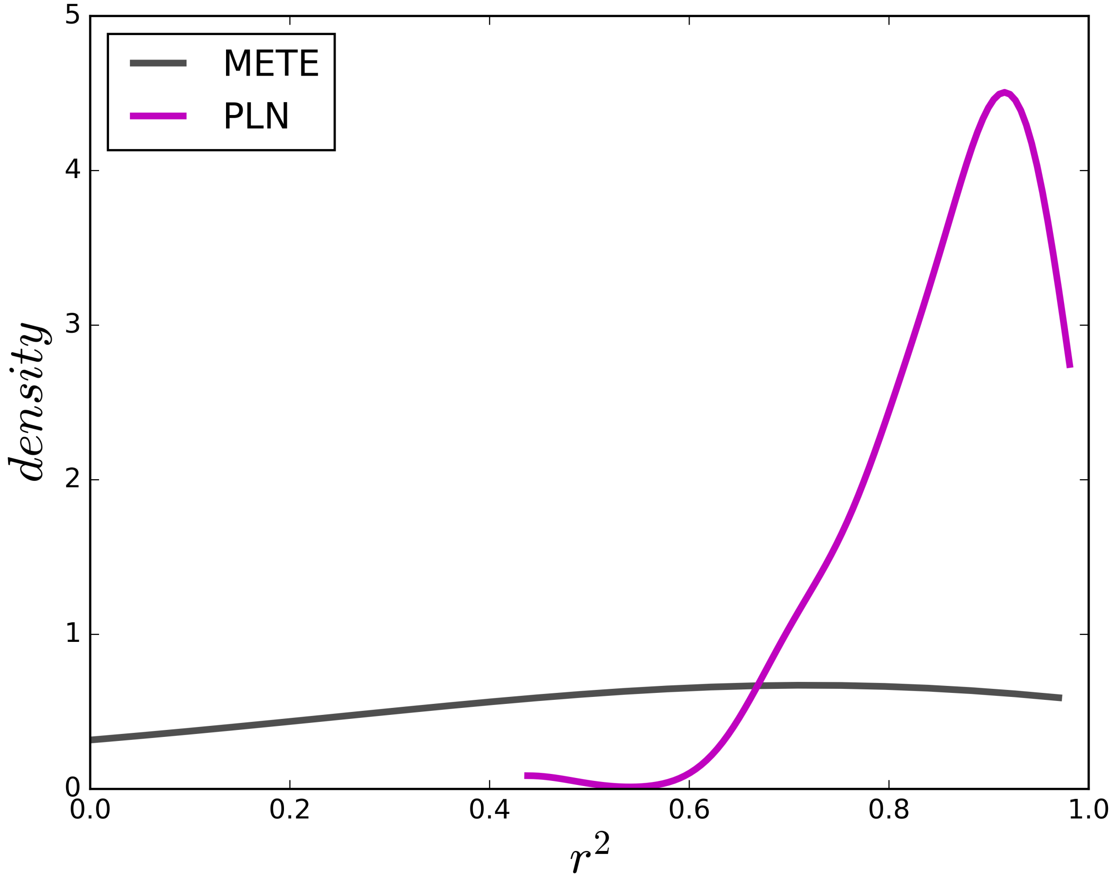

FIGURE S2 | Heat maps revealing noisy and complicated relationships of encounter to resource concentration in (left) well-mixed and (right) structured environments. In each plot, blue heat maps represent the results of models that included lock-and-key chemical complexity (complex molecules) and excluded active dispersal. Red heat maps represent the results of models that excluded chemical complexity (simple molecules) but included chemotaxis. Greater heat, i.e., areas within heat maps that have lighter colors, corresponds to a greater number of model results. Models with lowest encounter (blue heat maps) and highest encounter (red heat maps), occupy opposite ends of the relationship, and are characterized by markedly different microscale conditions.

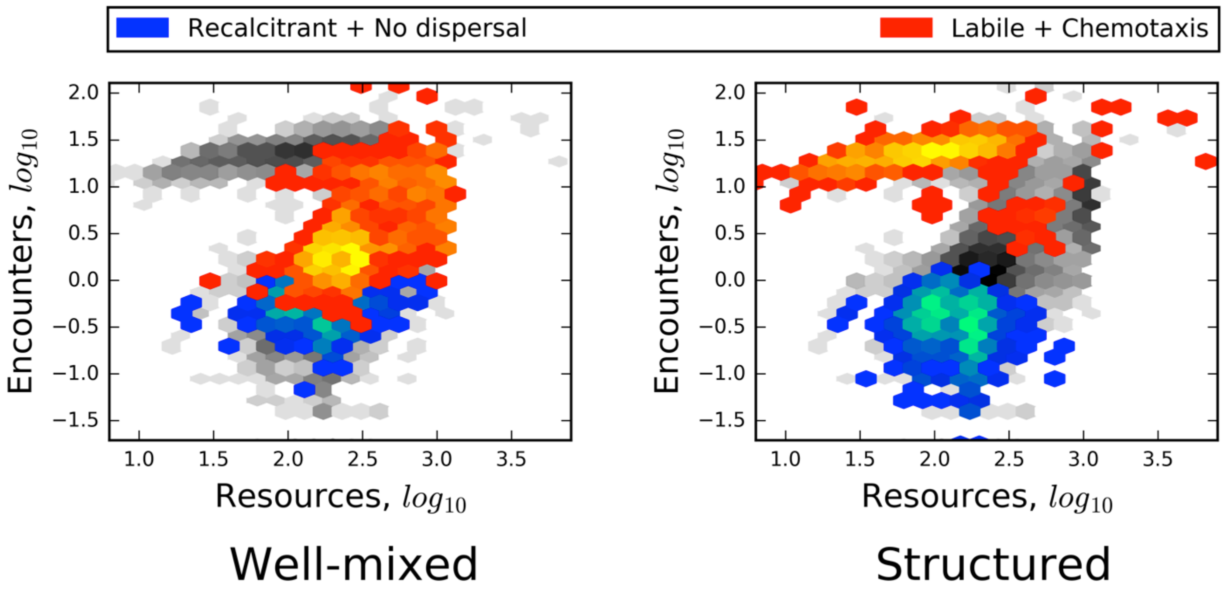

FIGURE S3 | Heat maps revealing no relationship of encounter to resource supply in (left) well-mixed and (right) structured environments. In each plot, blue heat maps represent the results of models that included lock-and-key chemical complexity (complex molecules) and excluded active dispersal. Red heat maps represent the results of models that excluded chemical complexity (simple molecules) but included chemotaxis. Greater heat, i.e., areas within heat maps that have lighter colors, corresponds to a greater number of model results. Models with lowest encounter (blue heat maps) and highest encounter (red heat maps), occupy opposite ends of the relationship, and are characterized by markedly different microscale conditions.

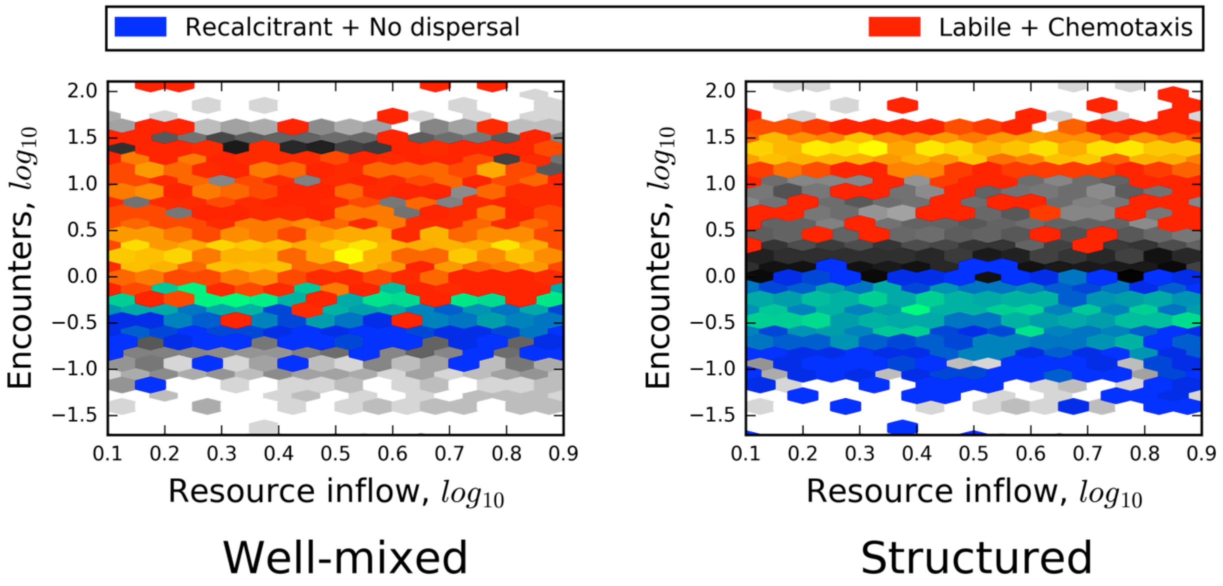

Supplement: Supplementary file 3 [file Image1.PDF]
